# Non-synchronous Structural and Functional Dynamics During the Coalescence of Two Distinct Soil Bacterial Communities

**DOI:** 10.3389/fmicb.2019.01125

**Published:** 2019-05-29

**Authors:** Xiaogang Wu, Ji Li, Mengmeng Ji, Qiaoyu Wu, Xinxin Wu, Yiming Ma, Weikang Sui, Liping Zhao, Xiaojun Zhang

**Affiliations:** State Key Laboratory of Microbial Metabolism, Joint International Research Laboratory of Metabolic & Developmental Sciences, School of Life Sciences and Biotechnology, Shanghai Jiao Tong University, Shanghai, China

**Keywords:** soil bacterial community, reciprocal inoculation, biological function, community coalescence, functional redundancy

## Abstract

Soil is a unique environment in which the microbiota is frequently subjected to community coalescence. Additions of organic fertilizer and precipitation of dust induce coalescent events in soil. However, the fates of these communities after coalescence remain uncharted. Thus, to explore the effects of microbiota coalescence, we performed reciprocal inoculation and incubation experiments in microcosms using two distinct soils. The soils were, respectively, collected from a cropland and an industrial site, and the reciprocal inoculation was performed as models for the incursion of highly exotic microbiota into the soil. After incubation under either aerobic or anaerobic conditions for two months, the soils were assayed for their bacterial community structure and denitrification function. According to the 16S rRNA gene sequencing results, the inoculated soil showed a significant shift in bacterial community structure after incubation—particularly in the industrial soil. The structures of the bacterial communities changed following the coalescence but were predicted to have the same functional potential, e.g., nitrogen metabolism, as determined by the quantification of denitrifying genes and nitrogen gas production in the inoculated soil samples, which showed values equivalent those in the original recipient soil samples regardless of inoculum used. The functional prediction based on the known genomes of the taxa that shifted in the incubated sample communities indicates that the high functional overlap and redundancy across bacteria acted as a mechanism that preserved all the metabolic functions in the soil. These findings hint at the mechanisms underlying soil biodiversity maintenance and ecosystem function.

## Introduction

Recent investigations have drawn attention to community coalescence, which is a newly coined term describing the phenomena of community-community encounters in which two or more entire communities (and their environments) interact and assemble into a coalescent community (Rillig et al., 2015, 2016a; Tikhonov, 2016; Rillig and Mansour, 2017). Because microbes are relatively small compared to their habitats, even small habitat volumes contain microbial communities with a great diversity of microbes. A mixing of exotic and resident microbiota can easily occur following additions of soil, litter, water flow and anthropogenic materials. Coalescence is likely an important factor for community assembly (Tikhonov, 2016) because the newly assembled community cannot easily be separated into different parts. Moreover, because the characteristics of the assembled community differ from those of the original communities, the significance of the effects of microbiota coalescence has recently been emphasized (Mansour et al., 2018) and may be fundamental for the generation of microbial communities (Rillig and Mansour, 2017).

Compared with other environments, soils harbor an unparalleled diversity of microorganisms (Fierer et al., 2012) and are uniquely suited for studying the coalescence of complex microbiota. Examples of the drivers of coalescence include the action of earthworms, the dynamics of soil aggregates, tillage, litter-fall, outplanting, flooding and the addition of materials containing existing microbial communities (such as stored biochar, manure or compost) (Rillig et al., 2016a). Human-associated pathways, including agricultural activities, can also accidentally transport exotic microbiota to soils (McNeill et al., 2011; Rillig et al., 2016a).

A number of investigations have already demonstrated that microbial coalescence is an important driving force in the assembly of soil microbial communities (Rillig et al., 2016b). An experiment performed with a mixture of soils from agricultural, forest and grassland sites to increase the diversity of soil microbes in the initial material and improve the ability of the soil to support the above-ground plants showed that the coalescence of soil communities promoted subsequent increases in plant biomass and an accompanying enhancement of microbial extracellular enzyme activities associated with nitrogen mineralization (Panke-Buisse et al., 2015; Rillig et al., 2016b). The reproducibility of the flowering phenotype across plant hosts suggests that microbiome mixtures can be selected that modify plant traits and coordinate changes in soil resource pools (Panke-Buisse et al., 2015; Rillig et al., 2016b). Another investigation using a culture-based method showed that adding sterilized leaves to soil led to a significantly different structure of the microbiota in leaf litter compared to leaf litter from which the endophyte community was not excluded (Osono, 2005). This outcome suggests that with the exception of nutrition, endophytes from the leaves contributed to the assembly of soil microbiota. In another experiment, root-colonizing microbiota in soil of *Dactylis glomerata* was observed to be similar to neighboring microbiota associated with *Centaurea maculosa* rather than with the typical root microbiota of *D. glomerata*, indicating the coalescence of root microbiota in the soil (Mummey et al., 2005). This finding was subsequently confirmed by Hausmann and Hawkes (2009). Thus, investigations into the coalescence of indigenous and invading communities are essential for understanding the mechanism by which soil microbiota is generated *in situ*.

Additionally, there has been growing interest in mixing foreign soil or other materials to improve soil-dressing technology in recent years (Swenson et al., 2000), especially in China (Zhou et al., 2003). However, little is known regarding the effects associated with the use of these technologies on soil ecosystems (Rillig et al., 2016a). In this study, we evaluated the ecological effects of the coalescence of two distinct bacterial communities from an agricultural soil and a severely contaminated industrial soil. This experiment may mimic the application of industrial waste to agricultural crop fields, where the introduction of polluted soil and their influences on the native soil microbiota and function is a serious concern. Thus, we set up microcosms and performed reciprocal inoculation using these two distinct soils. After two months of incubation, the bacterial compositions of the soil microcosms were examined, and the functional metagenomes were predicted based on 16S rRNA gene sequencing. The soil physicochemical parameters, nitrogen cycling-associated gene copy numbers, and nitrogen reduction function were also evaluated.

## Materials and Methods

### Soil Sampling

Two different soils were used in this investigation. The first soil sample (ACS) was collected from an industrial site in Baoshan (31°41′N, 121°46′E), Shanghai, where the soil has been severely contaminated by organic pollutants for many years, particularly aromatic compounds. The ACS soil samples were sampled using GeoProbe Systems (GeoProbe Inc., United States) at a depth of 150 cm. The parent material at the Baoshan site is primarily Yangtze River alluvial soil. The second soil sample (ONS) was obtained from cultivated cropland under optimized nitrogen fertilization and straw management practices in Quzhou (36°86′N, 115°02′E), Hebei Province. The ONS soil samples were collected from the top 20 cm using a sterile manual corer (10-cm diameter). The fields at the sampling site were planted on intensively managed agricultural soils typical of the North China Plain, where winter wheat–summer maize rotation is the dominant crop production system, and urea and ammonium (NH_4_^+^)-based fertilizers are the most commonly applied nitrogen fertilizers. The soil of the North China Plain is classified as a calcareous fluvo-aquic soil (Huang et al., 2013; Yang et al., 2017). The initial water-holding capacities (WHC) of the ONS and ACS soils were 38 and 49%, respectively. Samples of both soils were placed in sterile plastic self-sealing bags and kept on ice until being transported to the lab, as described previously (Xun et al., 2015). After sieving the soils, sterile water was added to maintain a constant moisture level (26 and 19% of field capacity for the ACS and ONS soils, respectively).

### Reciprocal Inoculation of the Two Different Soils

In the mixture groups, 3 g each of the ONS and ACS soils were reciprocally inoculated and mixed with 30 g each of the ACS and ONS soils. The mixtures were subsequently individually incubated for 2 months under aerobic and anaerobic conditions (the details of the reciprocal inoculation experimental design are described in Table 1). For the control groups, 30 g of either ONS or ACS soil was incubated for 2 months under the same aerobic and anaerobic conditions (Table 1). Each soil sample was vigorously stirred with a sterile glass rod in a 100-mL vial, which was subsequently sealed for incubation. The bottles for the aerobic management treatment were sealed directly under a laminar flow bench (Shanghai, China), while the air in the anaerobic management treatment bottles was replaced with helium using a pump ventilation system (Shanghai, China). The vials were incubated at 25°C in darkness, and each treatment was performed in triplicate.

**Table 1:** Experimental design of reciprocal inoculation.

Treatments^a^	Description
AintoOae	3 g ACS soil was inoculated into ONS soil and incubated for 2 months under aerobic condition.
AintoOan	3 g ACS soil was inoculated into ONS soil and incubated for 2 months under anaerobic condition.
OintoAae	3 g ONS soil was inoculated into ACS soil and incubated for 2 months under aerobic condition.
OintoAan	3 g ONS soil was inoculated into ACS soil and incubated for 2 months under anaerobic condition.
ACSae	30 g ACS soil was incubated for 2 months under aerobic condition.
ACSan	30 gram ACS soil was incubated for 2 months under anaerobic condition.
ONSae	30 gram ONS soil was incubated for 2 months under aerobic condition.
ONSan	30 gram ONS soil was incubated for 2 months under anaerobic condition.


*^a^Each treatment has three replicates.*

### Determination of Basic Soil Characteristics

The soil pH was measured with a pH meter (Mettler-Toledo, Switzerland) at a soil: water ratio of 1:2.5. Dissolved organic carbon (DOC) was determined using an Elab-TOC analyzer (Suzhou Elab Analytical Instrument Co., Ltd., China), and dissolved nitrogen (DN) was determined using a Shimadzu TN (total nitrogen) unit (Shimadzu Corporation, Japan).

### Analysis of the Soil Bacterial Community

DNA was extracted from 0.5 g of each soil sample with cetyltrimethylammonium ammonium bromide (CTAB), as described previously (Griffiths et al., 2000; Paulin et al., 2013). DNA quality was assessed based on the 260/280 nm absorbance ratio, which was measured using a BioDrop μLITE device (Biochrom, United Kingdom).

The DNA from 26 soil samples was sequenced, including 24 samples from the eight treatment groups (triplicates in each groups) and two samples from the original untreated soils (ONS and ACS) (Table 1 and Supplementary Table S1). The extracted DNA was used as a template to amplify the V3–V4 region of the 16S rRNA gene. The PCR operation, sequencing of the PCR amplicons and raw data analysis were performed in accordance with the protocol provided by Illumina for the MiSeq system (Part # 15044223 Rev. B; Illumina Inc., United States) as previously described with minor modifications (Qin et al., 2018). The preparation of the sequencing library, including DNA extraction and PCR amplification, was conducted as previously described (Qin et al., 2019). Specifically, the V3–V4 hypervariable region of the bacterial 16S rRNA gene was amplified from the genomic DNA using the universal primer set B341F/B785F (Li et al., 2018). The purified amplified products were sequenced using an Illumina MiSeq system (Qin et al., 2019).

### Bioinformatics and Sequencing Data Analysis

Both the forward and reverse ends of the read were trimmed at the base of which a Q value being less than 20. If the pair of reads overlapped by a minimum of 50 bp, they were merged into a complete read. Reads smaller than 399 bp and those with more than one expected error were discarded from the analysis (Edgar, 2010). The quality-filtered reads were dereplicated into unique sequences, sorted in decreasing abundance, and singletons were discarded. Representative non-chimeric operational taxonomic unit (OTU) sequences were subsequently obtained using the default UPARSE settings (Edgar, 2013), and additional reference-based chimera detection was performed using UCHIME (Edgar et al., 2011). The OTU table was generated after mapping the quality controlled reads to the non-chimeric OTUs using Usearch (Edgar, 2010) global alignment algorithm at a 97% cutoff in conjunction with filtering to exclude non-bacterial data.

Analysis of the bacterial community structure was performed using QIIME (Caporaso et al., 2010). The number of high-quality reads was greater than 10,000 for all samples. Therefore, the sequences of each samples were randomly re-extracted with 10,000 reads each time with 1,000 permutations for rarefaction and normalization to equalize the differences in sequencing depth using QIIME (Caporaso et al., 2010). The purpose of these multiple rarefactions was to ensure that the data features were preserved verifiably; the multiple permutations can reduce errors caused by randomness. The alpha diversity of each sample was calculated by determining the observed OTUs, the Shannon index, the Simpson index and the phylogenetic diversity (PD) whole-tree index according to the normalized data. Representative sequences for each OTU were built into a phylogenetic tree using FastTree and subjected to the Ribosomal Database Project (RDP) classifier to determine the phylogeny using a bootstrap cutoff of 80% (RDP database version 2.10). The phylogenetic tree and the relative abundance table of the representative OTU sequences were subsequently subjected to principal coordinate analysis (PCoA) (Lozupone and Knight, 2005). The data were graphed using GraphPad Prism (version 4.0 for Windows, GraphPad Software, San Diego, CA, United States) and MATLAB 2014a (The MathWorks INC., MA, United States). The statistical significance of the community structural similarity between different treatments was assessed by multivariate analysis of variance (MANOVA) using MATLAB 2014a (The MathWorks Inc., Natick, MA, United States). The statistical significance of the differences in microbial communities among the samples from different treatments was assessed by MANOVA.

### Prediction of Bacterial Community Function

The functional composition of the soil metagenome was predicted using Phylogenetic Investigation of Communities by Reconstruction of Unobserved States (PICRUSt) (Langille et al., 2013). The 16S rRNA gene data were referenced according to Greengenes 13.5, and the resulting data was used for predictive analysis by PICRUSt via the Kyoto Encyclopedia of Genes and Genomes (KEGG). The identified KEGG orthology (KO) pathways were subsequently sorted into functional categories based on the KEGG pathway subsystem hierarchy level 3 (according to KEGG module). The differences in the functional composition of the metagenomes in response to different soil management practices were compared using PICRUSt metagenome inferences, and the beta diversity analysis was conducted based on the Bray-Curtis distance. The differences in bacterial community biological function distributions were determined based on the predicted data categorized by function via the KOs. The statistical significance of the functional similarity between the samples from different treatments was assessed by MANOVA.

### Quantitation of Nitrogen Cycling Functional Genes After Reciprocal Inoculation

Quantitative real-time PCR was performed with a LightCycler 96 instrument (Roche, Switzerland) using SYBR Green as a fluorescent dye to determine the relative abundances of functional genes involved in nitrogen cycling, including nitrate reductase (*narG*) (Bru et al., 2007), nitrite reductase (*nirK* and *nirS*) (Henry et al., 2004; Throbäck et al., 2004), nitrous oxide reductase (*nosZ*) (Henry et al., 2006) and ammonia monooxygenase (*amoA*) genes (Yang et al., 2017) (Supplementary Table S2). One-way ANOVA was performed to test the variations in gene copy numbers (log transformed) among the different treatments.

### Determination of Soil Denitrification Function

Sodium nitrite (added concentration of nitrite-nitrogen (NO_2_^-^-N): 50 mg/kg soil delivered weight) and glucose (added concentration of DOC: 1 g/kg soil delivered weight) were added to the experimental vials containing 20 g of soil (the remaining soil in each vial was collected for soil index and microbiota determinations). Sodium nitrite and glucose were dissolved in 1 mL of sterile water and added to each vial. The headspace gas in all the vials was replaced with helium using a pump ventilation system (Shanghai, China). The vials were subsequently placed in a robotized incubation system for incubation and gas monitoring (Molstad et al., 2007) for a duration of 5 days. The kinetics of nitric oxide (NO), nitrous oxide (N_2_O), and dinitrogen (N_2_) formation in the vials was monitored accordingly (Molstad et al., 2007). Student’s *t*-tests were performed to evaluate the differences in the N_2_-N concentration after 100 h of incubation between the uninoculated and inoculated samples.

### Accession Numbers of the Sequence Data

The 16S rRNA gene sequences obtained in this study were submitted to the GenBank Sequence Read Archive (SRA) database of the National Center for Biotechnology Information (NCBI) under the accession numbers SRP153935 and SRP162142.

## Results

### Soil Properties Before and After Incubation

The ONS and ACS soils had significantly different properties in terms of the basic characteristic indices. Compared with the ACS soil, more DNA was extracted from the ONS soil and it had a higher nitrate concentration; however, the ACS soil had a much greater concentration of heavy metal and aromatic pollutants (Supplementary Table S3). After the reciprocal inoculation and incubation, a comparison of the soil parameters, including the pH and the carbon and nitrogen contents, revealed that inoculation with other soils did not significantly influence the recipient soils (Table 2 and Supplementary Table S4).

**Table 2:** Soil pH, DOC, and DN after incubation.

Treatments	pH	Dissolved organic carbon (DOC)	Dissolved nitrogen (DN)
			
		(mg/kg of soil)	(mg/kg of soil)
AintoOae	7.86 ± 0.016^a^	222 ± 26.7	51 ± 4.5
AintoOan	7.94 ± 0.036	251 ± 18.6	39 ± 6.8
ONSae	7.82 ± 0.004	236 ± 28.7	55 ± 5.2
ONSan	7.91 ± 0.054	255 ± 20.8	42 ± 7.1
OintoAae	8.22 ± 0.091	97 ± 10.4	10 ± 0.9
OintoAan	8.17 ± 0.147	215 ± 95.7	11 ± 4.1
ACSae	8.27 ± 0.100	83 ± 9.1	6 ± 1.6
ACSan	8.20 ± 0.167	311 ± 203.7	7 ± 3.9


*^a^Values (mean ± standard deviation) indicate each index.*

### Shift in Bacterial Community Structure During Incubation

The bacterial community in each sample was analyzed via high-throughput sequencing of the V3–V4 region of the 16S rRNA gene. A total of 1,287,005 usable high-quality sequences (673,788 unique sequences) were selected for subsequent analyses after being checked for quality, and 4,566 OTUs were obtained (median = 31,585 sequences, ranging from 11,074 to 58,294 sequences). The ONS soil had a higher alpha diversity index than did the ACS soil. The bacterial richness showed no obvious increase with the exotic soil inoculation (Table 3 and Supplementary Table S5). The bacterial richness of the ONS soil inoculated with ACS soil (AintoO) was more stable than that of ACS soil inoculated with ONS soil (OintoA); the alpha diversity of the former treatment was similar to that of the ONS soil. However, the observed number of OTUs in the OintoA treatments was lower than the observed number in the ACS soil, and the number of OTUs observed in the AintoO treatments was lower than the number observed in the ONS soil (Table 3). The number of OTUs in the AintoO treatments was more stable than the number of OTUs in the OintoA treatments after incubation (Supplementary Table S5).

**Table 3:** Alpha diversity indices of different treatments.

Treatments	Observed OTUs^a^	Shannon^b^	Simpson^c^	PD whole tree^d^
AintoOae	1947 ± 444^e^	8.90 ± 0.19	0.99 ± 0.001	109.4 ± 20.72
AintoOan	2021 ± 417	9.03 ± 0.12	1.00 ± 0.001	112.4 ± 18.73
ONSae	2206 ± 309	8.86 ± 0.24	0.99 ± 0.002	126.4 ± 13.05
ONSan	2241 ± 355	8.99 ± 0.25	0.99 ± 0.001	127.8 ± 15.46
ONSoriginal	2359	8.73	0.99	138.9
OintoAae	618 ± 378	6.94 ± 0.67	0.97 ± 0.007	50.1 ± 18.71
OintoAan	697 ± 153	7.53 ± 0.25	0.98 ± 0.001	58.9 ± 9.59
ACSae	1002 ± 29	5.63 ± 0.06	0.92 ± 0.004	70.1 ± 1.94
ACSan	1156 ± 40	5.61 ± 0.21	0.90 ± 0.017	80.2 ± 3.06
ACSoriginal	789	5.71	0.91	62.8


*^a^Detected OTU (operational taxonomic units) number. ^*b*^Shannon entropy of counts, default in bits. ^*c*^Simpson’s index. ^*d*^Phylogenetic diversity. ^*e*^Values (mean ± standard deviation) indicate each index.*

**FIGURE 1:**
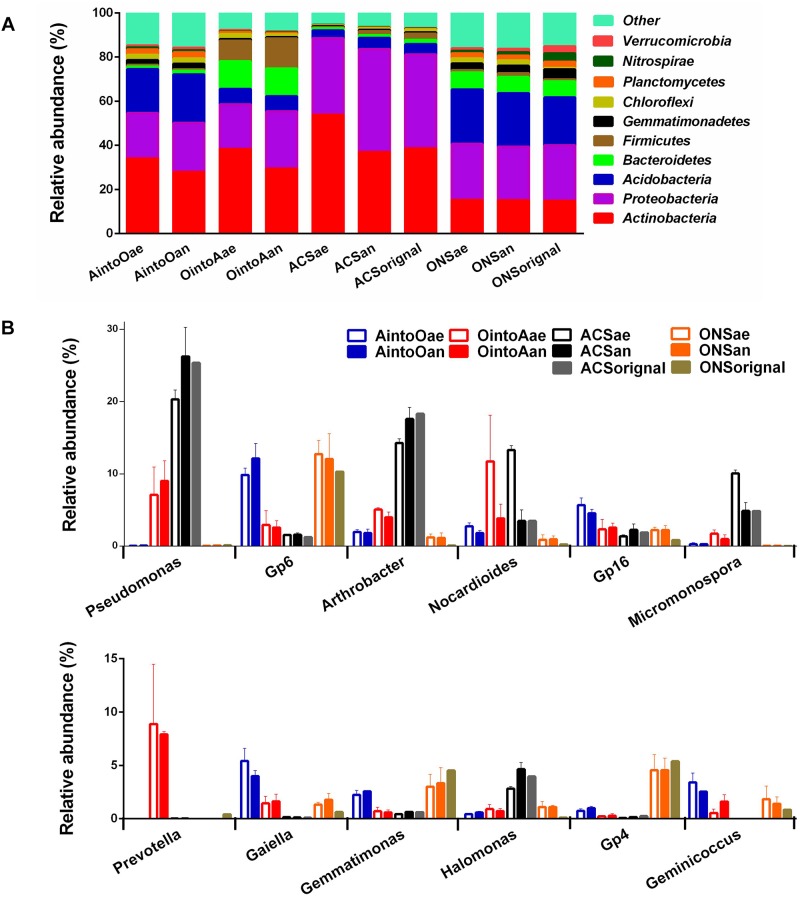
Community compositions of different treatments after incubation. **(A)** Relative abundances of predominant phyla in the samples of different treatments. **(B)** Relative abundance of predominant genera in the samples of different treatments.

Distinct taxonomic compositions were observed in the initial soil samples used in this study. At the phylum level (Figure 1A), the ACS soil had greater abundances of Proteobacteria and Actinobacteria, while the ONS soil had greater abundances of Acidobacteria and Bacteroidetes. After incubation for two months, the ONS soil inoculated with ACS presented increased amounts of Proteobacteria but a reduction in Bacteroidetes. In contrast, the ACS soil inoculated with ONS presented increased amounts of Firmicutes and Bacteroides. At the genus level (Figure 1B), the ACS soil had high abundances of *Pseudomonas*, *Arthrobacter*, *Nocardioides*, *Micromonospora*, and *Halomonas*, whereas the ONS soil had high abundances of *Gp6*, *Gp16*, *Gemmatimonas*, *Gp4*, and *Geminicoccus*. Other than the high abundance of *Gp6*, *Gp16*, *Geminicoccus*, and *Gemmatimonas*, three genera, *Gaiella*, *Nocardioides* and *Arthrobacter*, which are common bacteria in agricultural soils (Topp et al., 2000; Aislabie et al., 2005; Eo et al., 2015), were enriched in the AintoO treatment soils. In contrast, the OintoA-treated soils exhibited increases in the abundances of *Pseudomonas*, *Nocardioides*, *Prevotella*, *Arthrobacter*, *Gp6*, *Gp16*, *Gaiella*, *Geminicoccus*, and *Micromonospora*. Moreover, the abundances of five genera, *Gp6*, *Gp16*, *Gaiella*, *Geminicoccus*, and *Micromonospora*, which have been reported as indigenous bacteria in aromatic and organic compound-contaminated sites (Mcalpine et al., 2008; Franzetti et al., 2011; Pérez-Leblic et al., 2012), increased in the soils in response to the OintoA treatment. Additionally, while *Prevotella* and *Gaiella* OTUs were rare in both ONS and ACS soil, both OTUs were abundant in the OintoA and AintoO treatments, respectively (Figure 1B).

**FIGURE 2:**
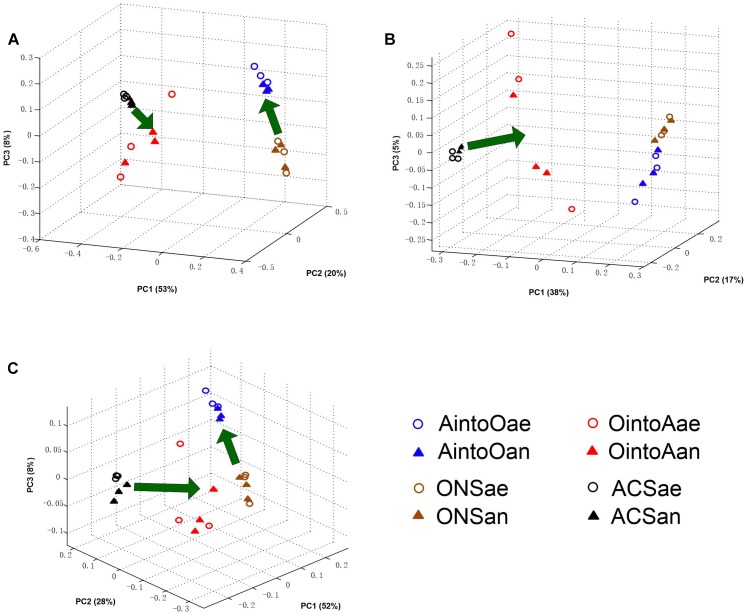
Community coalescence of the soil bacterial communities after reciprocal inoculation. **(A)** Bray-Curtis PCoA of the soil bacterial community structure based on the OTU data. **(B)** Unweighted UniFrac PCoA of the soil bacterial community structure based on the OTU data. The percentage of the variation explained by the plotted PCs is shown in parentheses. **(C)** Weighted UniFrac PCoA of the soil bacterial community structure based on the OTU data. The percentage of the variation explained by the plotted PCs is shown in parentheses. The percentage of the variation explained by the plotted principal coordinates (PCs) is shown in parentheses.

The PCoA results obtained using the Bray-Curtis distance and both weighted and unweighted UniFrac distances revealed the same pattern (Figure 2A–C). Reciprocal inoculation resulted in a significant alteration in community structure between the uninoculated and inoculated samples after 2 months of incubation. The MANOVA test based on Bray-Curtis distance and both weighted and unweighted UniFrac distances showed that difference in community structure between the inoculated and uninoculated treatments was significant (*P* < 0.001).

### Response of Bacterial Functional Genes to the Reciprocal Inoculations

**FIGURE 3:**
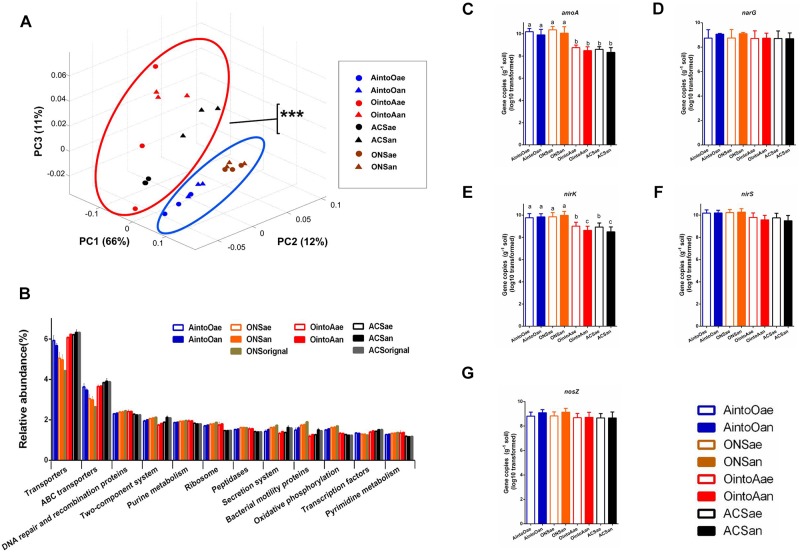
Predicted bacterial community biological function in soil treatments based on PICRUSt, according to both 16S rRNA gene sequencing data and the copy number of genes involved in nitrogen cycling within a gram of soil after long-term incubation. **(A)** PCoA of soil bacterial biological functions based on the Bray-Curtis distance according to KEGG module predictions in conjunction with the 16S rRNA gene sequencing data. The percentage of the variation explained by the plotted PCs is shown in parentheses. MANOVA analysis indicated significant difference between the circled clusters (^∗∗∗^*P* < 0.001). **(B)** Relative abundance of principal functions by PICRUSt predictions via a KEGG subsystem analysis of samples. All of the data are shown as the means ± standard deviations. The columns and error bars represent the means and standard deviations, respectively. **(C–G)** Copy number of genes involved in nitrogen cycling in the samples. **(C)** *amoA* gene, **(D)** *narG* gene, **(E)** *nirK* gene, **(F)** *nirS* gene, and **(G)** *nosZ* gene. One-way ANOVA was used to analyze variations among all the treatments. Different small letters indicate significant differences at the *P* < 0.05 level, and no significant differences were observed among the soil incubation samples for the *narG*, *nirS*, and *nosZ* genes.

To evaluate the bacterial functions in the soil samples before and after incubation, we predicted the functional gene content based on the 16S rRNA gene sequencing data using PICRUSt. The PCoA plots based on the PICRUSt data showed that the functional composition of the AintoO samples was most similar to that of the ONS soil samples and that the functional composition of the OintoA samples was most similar to that of the ACS soil samples (Figure 3A). The principal functions of the samples involved components such as transporters, ABC transporters, DNA repair and recombination proteins, two-component system proteins and processes such as purine metabolism (Figure 3B). The ONS samples had an increased abundance of DNA repair and recombination proteins; ribosomes; peptidases; bacterial motility proteins; and proteins involved in purine metabolism, oxidative phosphorylation and pyrimidine metabolism. In contrast, the ACS samples had an increased abundance of transporters, ATP-binding cassette (ABC) transporters and transcription factors (Figure 3B). However, the relative abundances of principal functions did not shift obviously in the inoculated samples. These results demonstrate that although the inoculation slightly altered the abundances of bacterial functional genes, the bacterial functional metagenomes remained stable.

Using quantitative real-time PCR, we also analyzed the abundances of genes involved in nitrogen cycling in soil. The quantification results for the *amoA*, *narG*, *nirK*, *nirS*, and *nosZ* genes after 2 months of incubation showed that sample inoculation with a different community did not affect the copy numbers of these genes for either soil recipient (Figure 3C–G and Supplementary Table S6). However, the gene copy numbers for *amoA* and *nirK* genes were significantly different between the two soil types; the ONS soil samples had more copies of the *amoA* and *nirK* genes than did the ACS soil samples.

### Response of Denitrification to the Reciprocal Inoculations

**FIGURE 4:**
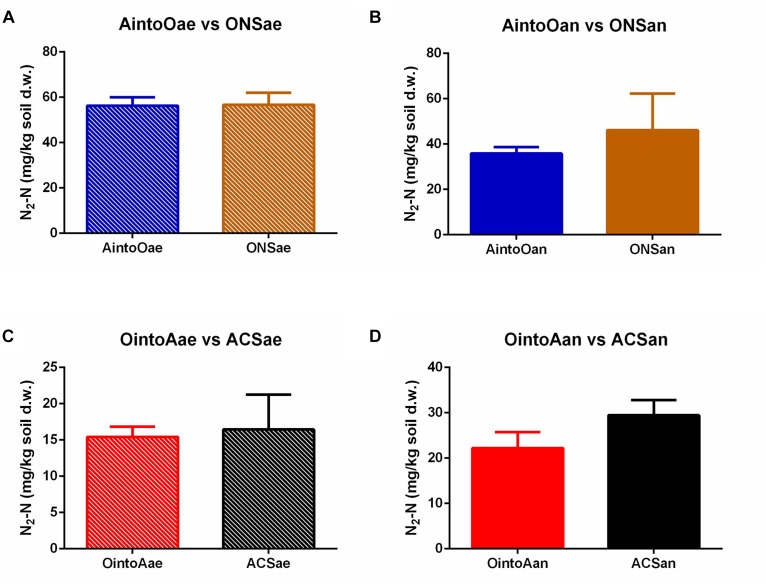
Dinitrogen content in different treatments after 100 h of kinetics monitoring. **(A)** AintoOae and ONSae. **(B)** AintoOan and ONSan. **(C)** OintoAae and ACSae. **(D)** OintoAan and ACSan. The bars indicate the means, and the error bars indicate the SD. No significant differences in the N_2_-N levels were observed between the inoculated and uninoculated soils.

The dynamics of the denitrification metabolism of the soil samples were measured by monitoring emitted gases using the robotized incubation system. The dynamics analysis of the emitted gaseous nitrogen showed that the denitrification activity in the reciprocally inoculated soil samples was unaltered compared with the uninoculated soils (Figure 4 and Supplementary Figure S1). Student’s *t*-tests indicated that there were no significant differences in the N_2_-N concentration after 100 h of monitoring between samples of AintoOae and ONSae (*P* = 0.9193), AintoOan and ONSan (*P* = 0.3413), OintoAae and ACSae (*P* = 0.7459), or OintoAan and ACSan (*P* = 0.0607). The results also showed that gas production trends were similar between the AintoO and ONS soil samples and the OintoA and ACS soils. Thus, the denitrification metabolism remained stable following the reciprocal inoculations.

## Discussion

Microbial community coalescence can result from the intermixing of diverse communities into a new environment or from one microbial community being mixed with another. Regarding the mixing ratios, coalescence processes can be differentiated by equal vs. unequal community mixing (Rillig et al., 2015). In our study, one microbiota was added to another at an unequal ratio to simulate the application of exotic soil into host soil. Two distinct recipient soils initially harbored different microbiota, and—as expected—the soil bacterial community structures shifted significantly after being inoculated with exotic bacteria. The MANOVA analysis showed that the introduced communities significantly influenced the host bacterial communities. Previous studies have shown that the biological invasion of exotic microbes resulting from soil management, i.e., fertilization and irrigation, influences the biodiversity and ecological function of soil recipients (Zhao et al., 2005; Watts et al., 2010; Zhang et al., 2012; Xiong et al., 2017). Recent studies have also demonstrated that species immigrating into a habitat may play an important role in shaping the habitat’s microbial community structure (Nemergut et al., 2013). This result is consistent with our findings: that inoculating communities instead of species can also reshape community structures, which is similar to organisms invading soils. In our study, some bacteria were enriched during the coalescence process. Specifically, some OTUs that were rare in both the ONS and ACS soils became abundant in the coalescent communities.

Our results indicated that bacterial richness was more stable in the agricultural soil than in the industrial soil (Supplementary Table S5). This result is probably due to the lower nutrient content and consequent stronger competition in the deep soil from the industrial site compared to the agricultural soil. It is well known that only species adapted to a specific habitat can survive; they would otherwise become extinct (Bazzaz, 1991; Woodward and Diament, 1991; Kolar and Lodge, 2001). Studies have also revealed the importance of phenotypic, physiological and biochemical traits (such as size, growth rate and resource utilization, respectively) on the performance of invasive species (Matz and Kjelleberg, 2005; Dawson et al., 2012; Mächler and Altermatt, 2012). Exotic bacteria must overcome the stability of the soil bacterial community to survive in a new habitat. In this study, we observed that the enriched exotic bacteria often dominated in soils whose environmental characteristics were the same as those of the recipient soils, including members of the genera *Gaiella* (Eo et al., 2015), *Nocardioides* (Topp et al., 2000), *Arthrobacter* (Aislabie et al., 2005), *Gp6* (Pérez-Leblic et al., 2012), *Gp16* (Pérez-Leblic et al., 2012), *Geminicoccus* (Franzetti et al., 2011), and *Micromonospora* (Mcalpine et al., 2008).

In this study, we also evaluated nitrite reduction to investigate the effect of coalescence when two distinct soils were reciprocally mixed in different ratios. Nitrite reduction, which eliminates the harmful compound nitrite from soil, is crucial to the soil nitrogen cycle (Malone and Stevens, 1998). Most bacterial genera that were predominant in our study samples, such as *Bradyrhizobium* (Fernandez et al., 2008), *Azoarcus* (Lee and Wong, 2014), *Thiobacillus* (Yang et al., 2018), and *Nitrosomonas* (Schmidt et al., 2004) are typical nitrite-reducing bacteria. The sum of the relative abundances of these bacteria remained stable during the incubation period (Supplementary Table S7). Some inoculated microorganisms may have a similar metabolic capacity for nitrite removal, although the abundances of different individual species varied after inoculation with exotic bacteria and incubation for the evaluated period of time. A robust correlation between community composition and ecological function has not been observed in various studies. The most direct reason for this lack of correlation has been attributed to the existence of functional redundancy (Rosenfeld, 2002). When a bacterial community structure is altered because of a disturbance, functional redundancy is very important for maintaining the function of the community (Baho et al., 2012). Functional redundancy refers to an overlap in the ecological functions of various species (Wohl et al., 2004; Comte and Giorgio, 2010). Many studies have attributed the stability (resistance and resilience) of soil ecosystems to the functional redundancy caused by intrinsic soil biodiversity, i.e., bacterial species decay is not always accompanied by bacterial functional decay (Chaer et al., 2009; Berga et al., 2012). Stable biological functions in conjunction with dynamic bacterial community structure have been reported in both studies of bioreactors (Xia et al., 2014; Wu et al., 2016; Qin et al., 2019) and of the human microbiome (Human Microbiome Project Consortium [HMPC], 2012; Lozupone et al., 2012; Bletz et al., 2016). For example, fecal and oral communities analyzed according to the 16S rRNA gene showed divergent community structure, whereas the results of shotgun metagenomics sequencing of the same samples showed obviously similar functional profiles (Human Microbiome Project Consortium [HMPC], 2012; Lozupone et al., 2012). In our study, we observed a reduction in the abundances of specific bacteria due to coalescence after mixing the host community with an exotic community, especially in the industrial soil. The abundances of some phylogenetically different bacteria increased to replace the indigenous bacteria. However, the incursion of exotic microbiota barely influenced the soil metabolism. Interestingly, these enriched bacteria were predicted to be functionally redundant with the replaced bacteria.

## Conclusion

In this study, the soil bacterial community structure shifted significantly in response to unequal mixing of two soils. The introduction of exotic microbiota impacted the soil bacterial community structure. Compared with the community structure observed in the industrial soil, the bacterial richness in the agricultural soil was less influenced by inoculation with the other soil, which may be due to a discrepancy in the nutritional conditions. Some bacteria taxa that were rare in both original soils became abundant after mixing and incubation, indicating that the new soil communities had coalesced. The soil microbiota is considered to be a self-organizing system that—due to functional redundancy—maintains the relative stability of biological function and adapts to disturbances caused by the incursion of exotic microbial communities.

## Author Contributions

XZ and XiaoW contributed conception and designed the study. XiaoW, JL, MJ, and QW performed the experiments and generated the data. XiaoW analyzed the data with assistance from XinW, YM, and WS. XiaoW wrote the first draft of the manuscript. XiaoW and XZ led the writing of the manuscript, with input from LZ.

## Conflict of Interest Statement

The authors declare that the research was conducted in the absence of any commercial or financial relationships that could be construed as a potential conflict of interest.
