# Supplementary material for: Non-synchronous Structural and Functional Dynamics During the Coalescence of Two Distinct Soil Bacterial Communities
Source: Front Microbiol. 2019 May 29;10:1125. doi: 10.3389/fmicb.2019.01125 (PMC6548817; doi:10.3389/fmicb.2019.01125)
Supplement: Supplementary file 1 [file Data_Sheet_1.PDF]

Supplementary material:

Main contents

Supplemental method
Supplemental tables: Table S1 to Table S7
Supplemental figure
Supplemental references

Supplemental method:

Quantitative real-time PCR was carried out on a Light cycler 96(Roche, Switzerland) using SYBR green as a fluorescent dye to determine the relative abundance of 16S ribosomal RNA (rRNA) (Nadkarni et al., 2002).

Soil properties were determined based on the following standard procedures used in China(Zhang and Gong, 2012). Concentrations of ammonia and nitrate in the extracts were determined by full wavelength spectrophotometry(Zhang and Gong, 2012). Soil nitrite was measured as described by Stevens, Laughlin, (1995).

The quality of sequencing run was also monitored with mock community controls of known species composition and relative abundance according to suggestions of the manufacturer’s instructions. The Sequence Read Archive (SRA) submission of SRP153935 will be released on 2019-08-14 or upon publication, whichever is first.

Supplemental tables:

Table S1 Sample messages in SRA

Samples name	SRR number	SRP number	Biosamples name in SRA
AintoOae-1	SRR7878487	SRP162142	BAS1U001SLD811
AintoOae-2	SRR7878486	SRP162142	BAS2U002SLD821
AintoOae-3	SRR7878489	SRP162142	BAS1U003SLD831
AintoOan-1	SRR7878488	SRP162142	BAS1V001SLD841
AintoOan-2	SRR7878491	SRP162142	BAS1V002SLD851
AintoOan-3	SRR7878490	SRP162142	BAS1V003SLD861
OintoAae-1	SRR7878493	SRP162142	BAS1W001SLD871
OintoAae-2	SRR7878492	SRP162142	BAS1W002SLD881
OintoAae-3	SRR7878495	SRP162142	BAS1W003SLD891
OintoAan-1	SRR7878494	SRP162142	BAS1X001SLD901
OintoAan-2	SRR7878485	SRP162142	BAS1X002SLD911
OintoAan-3	SRR7878484	SRP162142	BAS1X003SLD921
ONS original	SRR7878483	SRP162142	BAS1Y004SLD004
ACS original	SRR7878482	SRP162142	WXGBAS50
ACS-ae-1	SRR7526830	SRP153935	BAS1Q001SLD491
ACS-ae-2	SRR7526794	SRP153935	BAS1Q002SLD501
ACS-ae-3	SRR7526798	SRP153935	BAS1Q003SLD511
ACS-an-1	SRR7526799	SRP153935	BAS1R001SLD521
ACS-an-2	SRR7526781	SRP153935	BAS1R002SLD531
ACS-an-3	SRR7526821	SRP153935	BAS1R003SLD541
ONS-ae-1	SRR7526820	SRP153935	BAS1S001SLD551
ONS-ae-2	SRR7526795	SRP153935	BAS1S002SLD561
ONS-ae-3	SRR7526796	SRP153935	BAS1S003SLD571
ONS-an-1	SRR7526797	SRP153935	BAS1T001SLD581
ONS-an-2	SRR7526782	SRP153935	BAS1T002SLD591
ONS-an-3	SRR7526822	SRP153935	BAS1T003SLD601

Table S2 Primers and procedure used for real-time PCR analysis of various functional genes involved in N-cycling and 16S rRNA gene.

Gene	Primer	Sequence(5’-3’)[a]	Thermal profile
16S rRNA	Uni331F<br>Uni797R	TCCTACGGGAGGCAGCAGT<br>GGACTACCAGGGTATCTAATCCTGTT	95℃ for 180s<br>40 cycles×（95℃ for 30s,60℃ for 30s, 72℃ for 30s）， 80℃ for 5s, data was collected in the last step
amoA	amoA-1F<br>amoA-2R	GGGGTTTCTACTGGTGGT<br>CCCCTCKGSAAAGCCTTCTTC	95℃ for 180s,<br>40 cycles×（95℃ for 30s,60℃ for 30s, 72℃ for 30s）， 84℃ for 5s, data was collected in the last step
nirS	nirS cd3A<br>nirS R3cd	AACGYSAAGGARACSGG<br>GASTTCGGRTGSGTCTTSAYGAA	95℃ for 180s<br>40 cycles×（95℃ for 30s,58℃ for 30s, 72℃ for 30s）， 83℃ for 5s, data was collected in the last step
nirK	nirK-1040<br>nirK-FlaCu	GCCTCGATCAGRTTRTGGTT<br>ATCATGGTSCTGCCGCG	95℃ for 180s<br>40 cycles×（95℃ for 30s,58℃ for 30s, 72℃ for 30s）， 80℃ for 5s, data was collected in the last step
narG	narG-f<br>narG-r	TCGCCSATYCCGGCSATGTC<br>GAGTTGTACCAGTCRGCSGAYTCSG	95℃ for 180s<br>40 cycles×（95℃ for 30s,60℃ for 30s, 72℃ for 30s）， 80℃ for 5s, data was collected in the last step
nosZ	nosZ-2f<br>nsoZ-2r	CGCRACGGCAASAAGGTSMSSGT<br>CAKRTGCAKSGCRTGGCAGAA	95℃ for 180s<br>40 cycles×（95℃ for 30s,60℃ for 30s, 72℃ for 30s）， 84℃ for 5s, data was collected in the last step

[a] Degenerate bases: R=A/G, Y=C/T, M=A/C, K=G/T, S=C/G, W= A/T, H= A/C/T, B= C/G/T, V=A/C/G, D=A/G /T and N=A/C/G/T.

Table S3 Basic characteristic index of two different kinds of soil

	ONS original	ACS original
DNA quantity[a] (μg/g of soil)	26.5±4.35[b]	1.8±0.56
Water holding capacity	38%	49%
Soil moisture content	19%	26%
pH[a]	7.56±0.215	7.95±0.08
Dissolved organic carbon[a] (mg/kg of soil)	48.4±6.8	58±7
NH_4^+-N (mg/kg of soil)	6.2±2.27	1.8±0.10
NO_3^--N (mg/kg of soil)	40.5±2.32	1.9±0.51
NO_2^--N (mg/kg of soil)	0.09±0.03	0.01±0.01
Naphthalene (mg/kg of soil)	-	5.6
Anthracene (mg/kg of soil)	-	8.3
Benz(a)anthracene (mg/kg of soil)	-	33.9
Chrysene (mg/kg of soil)	-	7.7
Benzo(a)pyrene (mg/kg of soil)	-	6.1
Indeno(1,2,3-cd)pyren (mg/kg of soil)	-	11.6
Benzo[b]fluorathene (mg/kg of soil)	-	22.3
Dibenz[a,h]anthracene (mg/kg of soil)	-	1

[a]Values showed significant difference ($P < 0.001$) between ONS soil and ACS soil.

[b]Values (mean ±standard deviation) indicate the absolute amount of each characteristic

-Not detected.

Table S4 Statistics of the student's t test for discrimination of soil pH, dissolved organic carbon and dissolved nitrogen.

	pH	Dissolved organic carbon	Dissolved nitrogen
AintoOae vs ONSae	P= 0.4326<br>Not significant	P=0.5721<br>Not significant	P= 0.3160<br>Not significant
AintoOan samples ONSan	P= 0.5174<br>Not significant	P= 0.8177<br>Not significant	P= 0.6057<br>Not significant
OintoAae vs ACSae	P= 0.6278<br>Not significant	P=0.1567<br>Not significant	P= 0.3400<br>Not significant
OintoAan vs ACSan	P= 0.8467<br>Not significant	P= 0.3768<br>Not significant	P= 0.3848<br>Not significant

Table S5 Statistics of the student's t test for discrimination of alpha diversity

	Observed OTUs	Shannon	Simpson	PD whole tree
AintoOae vs ONSae	P=0.4476<br>Not significant	P= 0.8597<br>Not significant	P= 0.7059<br>Not significant	P= 0.7059<br>Not significant
AintoOan samples ONSan	P= 0.5236<br>Not significant	P= 0.7925<br>Not significant	P= 0.4539<br>Not significant	P= 0.4539<br>Not significant
OintoAae vs ACSae	P= 0.1545<br>Not significant	P= 0.0279<br>*<br>Significant	P= 0.0008<br>***<br>Significant	P= 0.0008<br>***<br>Significant
OintoAan vs ACSan	P= 0.0074<br>**<br>Significant	P= 0.0005<br>***<br>Significant	P=0.0014<br>**<br>Significant	P=0.0214<br>*<br>Significant

Table S6 Number of gene copies in a gram soil samples

Treatments	16SrDNA	amoA	narG	nirK	nirS	nosZ
AintoOae	$6.71\times10^{11}\pm4.85\times10^{11}$	$1.81\times10^{10}\pm1.25\times10^{10}$	$1.18\times10^{9}\pm1.53\times10^{9}$	$7.5\times10^{9}\pm6.46\times10^{9}$	$1.79\times10^{10}\pm1.28\times10^{10}$	$7.93\times10^{8}\pm6.43\times10^{8}$
AintoOan	$1.10\times10^{12}\pm3.15\times10^{11}$	$1.11\times10^{10}\pm8.62\times10^{9}$	$1.17\times10^{9}\pm1.65\times10^{8}$	$7.96\times10^{9}\pm4.51\times10^{9}$	$1.74\times10^{10}\pm8.97\times10^{9}$	$1.37\times10^{9}\pm7.87\times10^{8}$
ONS-ae	$7.28\times10^{11}\pm5.57\times10^{11}$	$2.67\times10^{10}\pm1.98\times10^{10}$	$1.22\times10^{9}\pm1.63\times10^{9}$	$9.22\times10^{9}\pm7.97\times10^{9}$	$1.97\times10^{10}\pm1.40\times10^{10}$	$8.24\times10^{8}\pm6.81\times10^{8}$
ONS-an	$1.45\times10^{12}\pm3.15\times10^{11}$	$1.77\times10^{10}\pm1.48\times10^{10}$	$1.29\times10^{9}\pm3.06\times10^{8}$	$1.16\times10^{10}\pm7.60\times10^{9}$	$2.15\times10^{10}\pm1.33\times10^{10}$	$1.60\times10^{9}\pm1.09\times10^{9}$
OintoAae	$3.85\times10^{11}\pm2.64\times10^{11}$	$6.25\times10^{8}\pm2.81\times10^{8}$	$8.70\times10^{8}\pm8.47\times10^{8}$	$1.27\times10^{9}\pm9.84\times10^{8}$	$7.99\times10^{9}\pm5.79\times10^{9}$	$5.83\times10^{8}\pm3.94\times10^{8}$
OintoAan	$1.04\times10^{11}\pm1.05\times10^{11}$	$3.80\times10^{8}\pm3.21\times10^{8}$	$7.52\times10^{8}\pm7.69\times10^{8}$	$5.57\times10^{8}\pm5.15\times10^{8}$	$5.09\times10^{9}\pm5.06\times10^{9}$	$7.09\times10^{8}\pm7.11\times10^{8}$
ACS-ae	$3.70\times10^{11}\pm2.74\times10^{11}$	$4.37\times10^{8}\pm2.10\times10^{8}$	$8.50\times10^{8}\pm7.94\times10^{8}$	$1.05\times10^{9}\pm8.01\times10^{8}$	$7.34\times10^{9}\pm5.32\times10^{9}$	$5.67\times10^{8}\pm3.76\times10^{8}$
ACS-an	$8.21\times10^{10}\pm8.83\times10^{10}$	$3.01\times10^{8}\pm3.14\times10^{8}$	$7.52\times10^{8}\pm8.45\times10^{8}$	$4.55\times10^{8}\pm4.89\times10^{8}$	$4.85\times10^{9}\pm5.49\times10^{9}$	$7.20\times10^{8}\pm8.19\times10^{8}$

[a] Values (mean ± standard deviation) indicate each index.

Table S7 Relative abundance of denitrification genus (%)

Treatments	*Bradyrhizobium*[a]	*Azoarcus*	*Thiobacillus*	*Nitrosomonas*	Total
AintoOae	0.072±0.019	0.001±0.001	0.012±0.017	0.006±0.002	0.090±0.027
AintoOan	0.074±0.023	0.005±0.004	0.051±0.041	0.005±0.008	0.134±0.053
ONS-ae	0.191±0.110	0.001±0.001	-	0.047±0.044	0.238±0.154
ONS-an	0.154±0.107	0.003±0.004	-	0.033±0.037	0.190±0.143
ONS-original	0.041	-	0.009	0.050	0.099
OintoAae	0.003±0.006	-	0.381±0.481	-	0.384±0.417
OintoAan	0.030±0.045	-	0.002±0.002	0.010±0.018	0.042±0.036
ACS-ae	0.008±0.005	-	0.017±0.002	-	0.025±0.007
ACS-an	0.003±0.004	0.001±0.001	0.050±0.002	0.001±0.001	0.055±0.007
ACS-original	0.009	-	0.054	-	0.063

[a] Genera such as *Pseudomonas* also possess denitrification bacterium, but most species of Pseudomonas are not denitrification bacteria.

[b] Values (mean ± standard deviation) indicate each index.

-Not detected.

Supplemental figure

Figure S1 Dynamics of gaseous nitrogen by robot reincubation. (A) Nitric oxide-nitrogen (NO-N) concentration dynamics. (B) Nitrous oxide-nitrogen (N2O-N) concentration dynamics. (C) Dinitrogen-nitrogen (N2-N) concentration dynamics. All the data are shown as the means and ranges. d.w.: delivered weight. There are no significant differences of N2-N after 100 hours incubation between inoculated and non-inoculated soil. ns: not significant (student's t test).

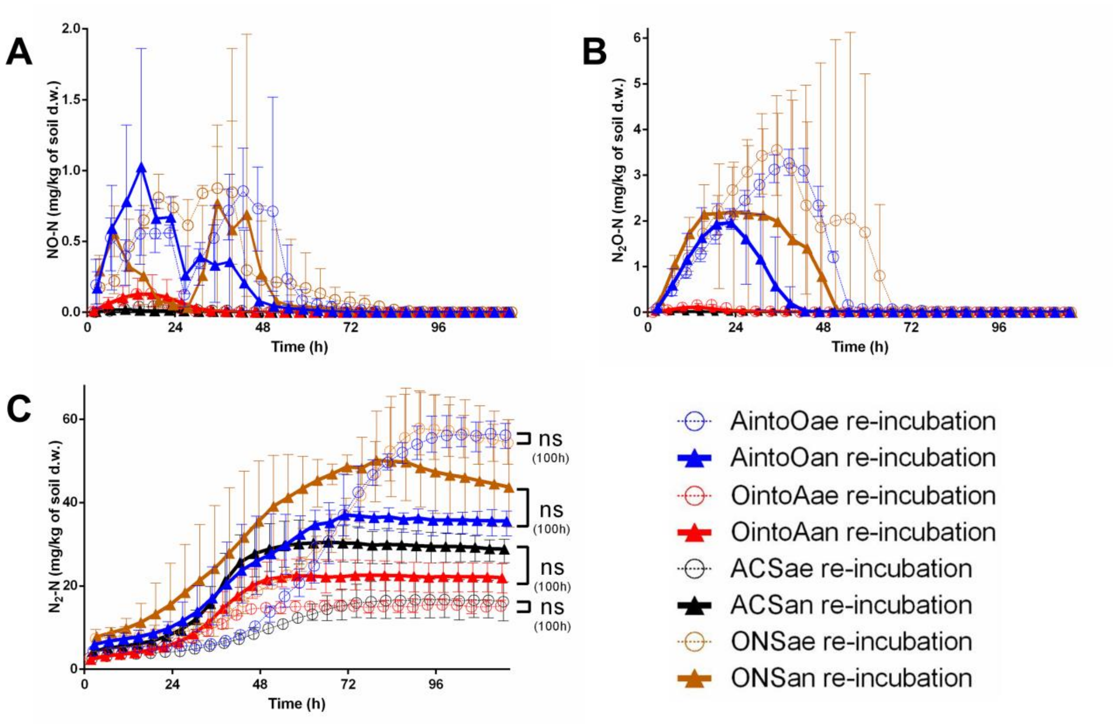

Supplemental references:

Nadkarni, M. A., Martin, F. E., Jacques, N. A., and Hunter, N. (2002). Determination of bacterial load by real-time PCR using a broad-range (universal) probe and primers set. *Microbiology*. 148, 257-266. doi:10.1099/00221287-148-1-257

Stevens, R. J., and Laughlin, R. J. (1995). Nitrite Transformations during Soil Extraction with Potassium Chloride. *Soil Science Society of America Journal*. 59, 933-938.

Zhang, G., and Gong, Z.: Soil Survey Laboratory Methods (in Chinese). Beijing , China : Science Press, (2012)
